# Supplementary material for: Violations of Löwenstein's rule in zeolites
Source: Chem Sci. 2017 Sep 5;8(11):7483–91. doi: 10.1039/c7sc02531a (PMC5676096; doi:10.1039/c7sc02531a)
Supplement: Supplementary file 1 [file SC-008-C7SC02531A-s001.pdf]

Electronic Supplementary Material (ESI) for Chemical Science.
This journal is © The Royal Society of Chemistry 2017

Supporting Information: Violations of Löwenstein's rule in zeolites

Rachel E. Fletcher, Sanliang Ling and Ben Slater*

Department of Chemistry, University College London, 20 Gordon Street, London

WC1H 0AJ, UK

*Email: b.slater@ucl.ac.uk

Index

**S1**	**Comparison of PBE with revPBE, BLYP (CP2K) and PBE (CASTEP) fro H-SSZ-13**	**2**
**S2**	**PBE0 calculations (H-SSZ-13)**	**2**
**S3**	**VDW corrected calculations (H-SSZ-13)**	**3**
**S4**	**DFT data for Li-SSZ-13 Si/Al = 17**	**4**
**S5**	**Global minimum structure for Li-SSZ-13 Si/Al = 17**	**4**
**S6**	**Global minimum structures for Na-SSZ-13 at Si/Al = 11, 8**	**5**
**S7**	**Table of framework topologies included in this study**	**6**
**S8**	**Non-Löwensteinian global minimum structures according to DFT for RHO, LTA, ABW**	**7**
**S9**	**Protonated MOR calculations**	**7**
**S10**	**SS MAS ^{29}Si NMR Data**	**8**
**S11**	**Vibrational frequency data**	**9**
**S12**	**Zero Point Energy Calculations and Thermodynamics Calculations**	**11**
**S13**	**CP2K example input file**	**12**
**S14**	**Coarse-grain sifting for potential low-energy structures using GULP**	**14**
**S15**	**References**	**14**

S1 The relative of stabilities of a collection of high-, mid-, and low-energy 2 Al per unit cell (Si/Al = 17) H-SSZ-13 (CHA) configurations, geometry optimized using CP2K with PBE, BLYP and revPBE functionals, and CASTEP at the PBE level of theory.[1–5]

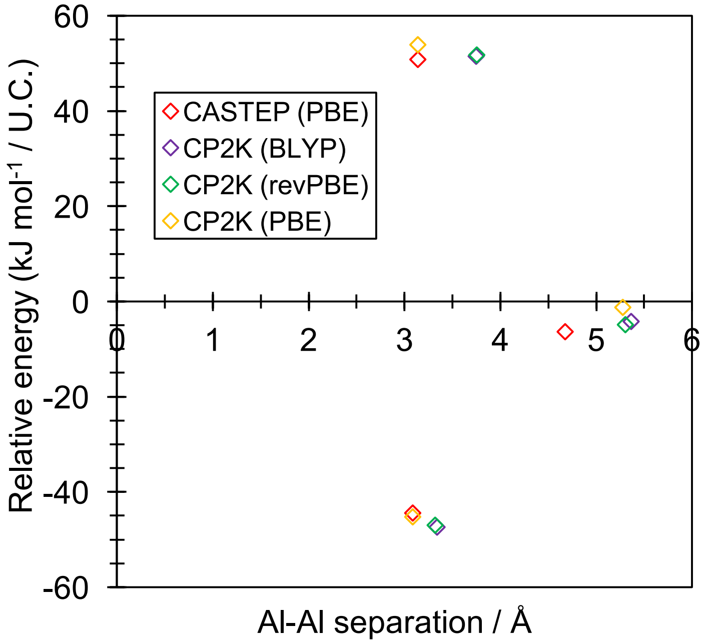

Energy differences between the three frameworks appear to be consistent. Discrepancies in Al-Al separation for the same models between the different levels of theory is due to differences in framework density following geometry optimization.

S2 Correlation between the relative energies of the 12 most stable 2 Al per unit cell (Si/Al = 17) H-SSZ-13 (CHA) structures, calculated using standard PBE and PBE0 hybrid functional implemented in CP2K using auxiliary density matrix methods (ADMM).[6,7]

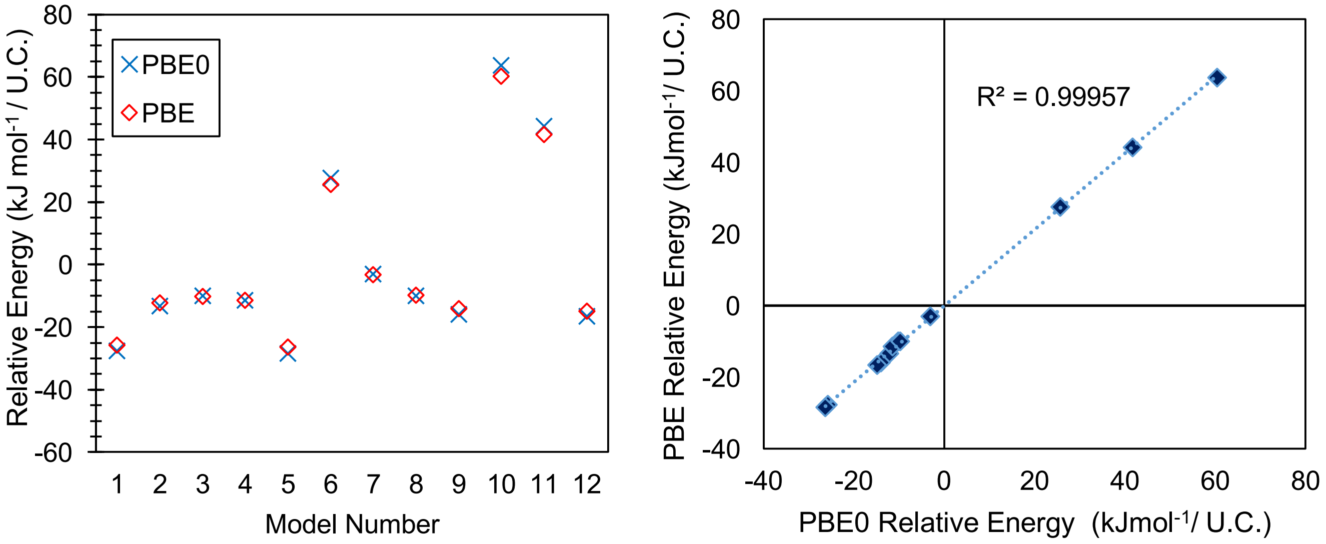

The PBE0 data points are single point energies performed on the PBE relaxed configurations. Both figures illustrate that there is good correlation between data obtained using the PBE functional, and the more sophisticated, more computationally demanding, hybrid PBE0 functional, such that many of the points shown on the graph above are in fact coincidental. Clearly, the PBE data captures the qualitative and quantitative differences between different configurations.

S3 Correlation between the relative energies of the 12 most stable 2 Al per unit cell (Si/Al = 17) H-SSZ-13 (CHA) structures, calculated using standard PBE and Van der Waals corrected functionals vdW-DF2 and PBE+D3.

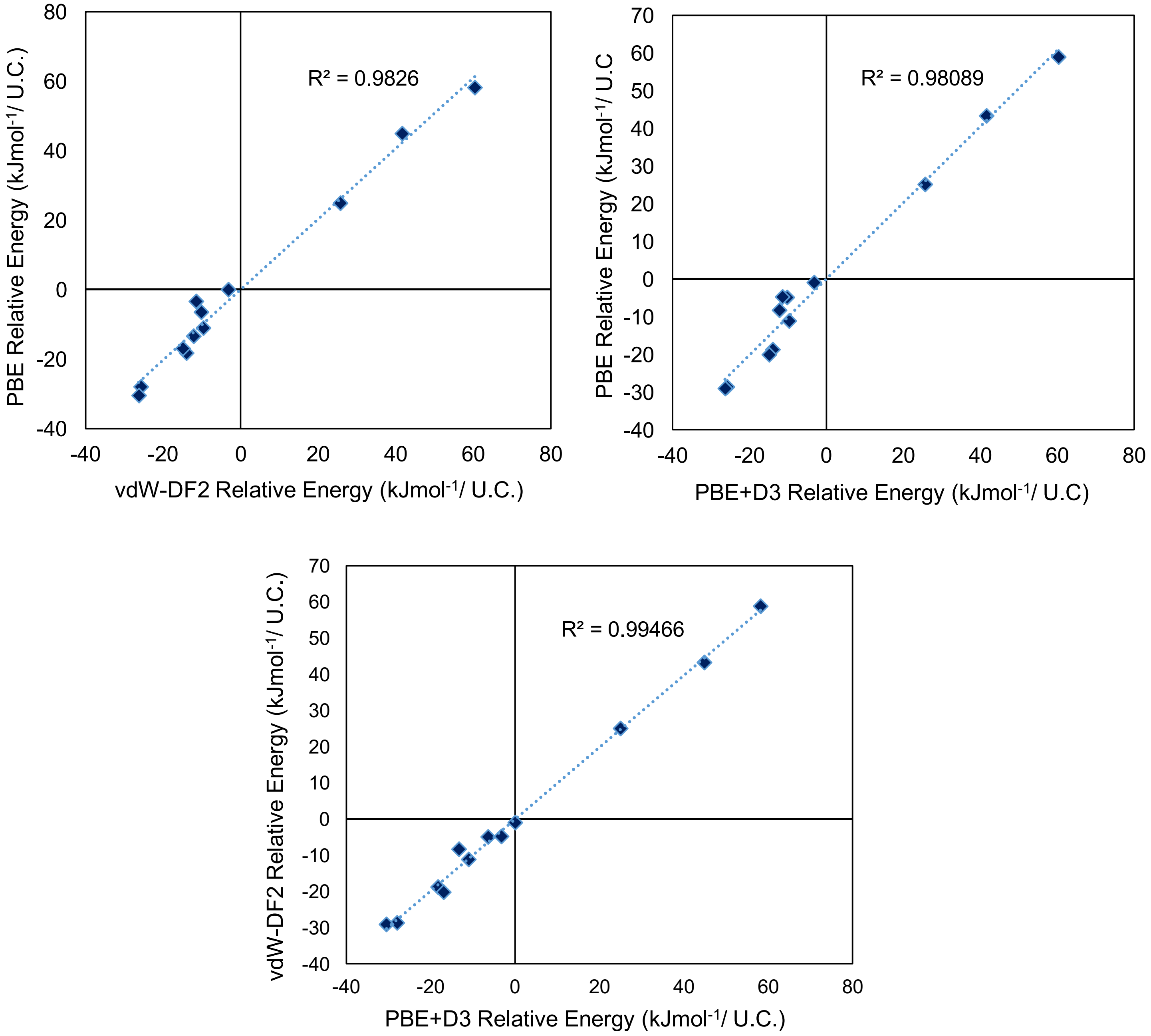

Correlation plots for PBE functional compared with A) vdW-DF2 and B) PBE+D3, Graph C) is a correlation plot of the relative energies from both vdW-DF2 and PBE+D3. All plots show good correlation between the standard PBE functional and vDW corrected functionals.

S4 Relative energy dispersion (kJmol^{-1} per U.C.) against framework aluminium separation for Li-SSZ-13 Frameworks possessing non-Löwensteinian ordered aluminium atoms (-Al-O-Al-) are shown in blue.

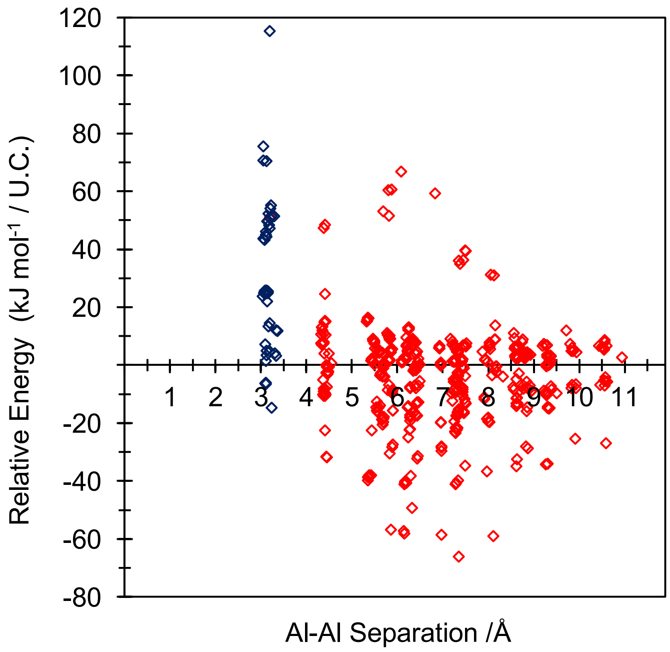

S5 Global minimum structure according to DFT for Li-SSZ-13 with 2 Al per unit cell (Si/Al = 17). A Löwensteinian structure with Li^{+} cations above and below the planes of the six-rings. Si are shown in yellow, O in red, Al in light blue and Li in dark blue.

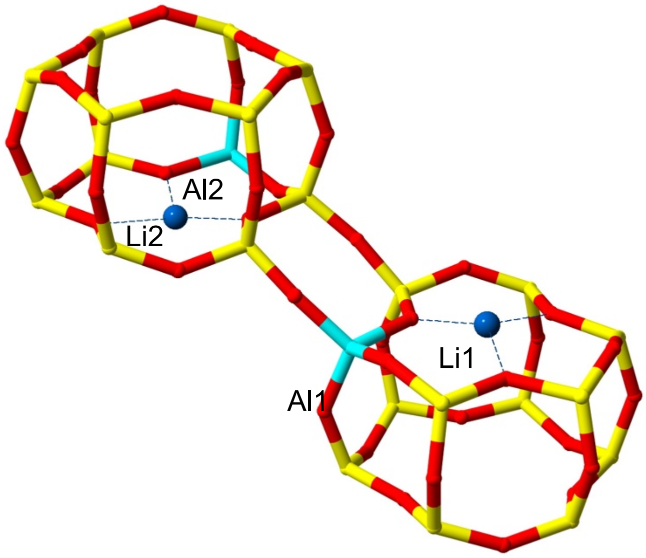

S6 Löwensteinian global minimum structures according to DFT for Na-SSZ-13 with 3 Al per unit cell (Si/Al = 33) originating from the H-SSZ-13 2 Al per unit cell NL and L global minimum structures, and 4 Al per unit cell (Si/Al = 32) originating from the H-SSZ-13 NL global minimum structure.
A) NL 3Al per unit cell Na-SSZ-13 B) L 3 Al per unit cell Na-SSZ-13 C) 4 Al per unit cell Na-SSZ-13

A

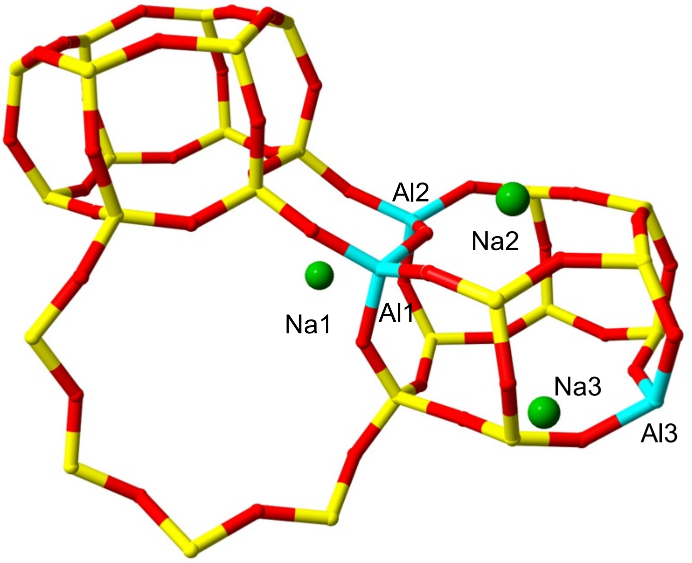

B

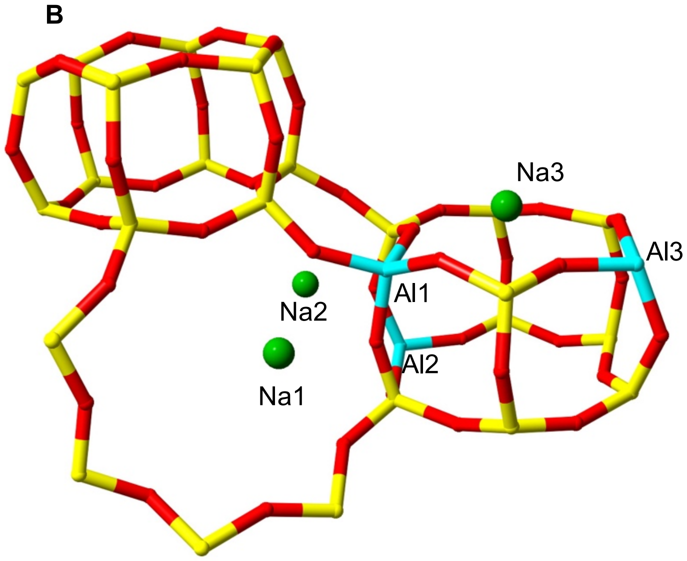

C

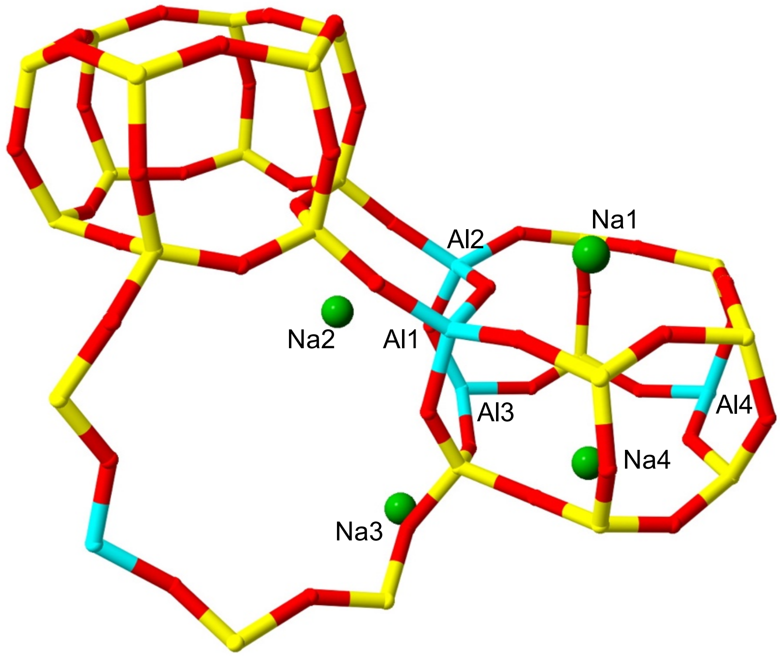

S7 Framework topologies included in this study, their corresponding framework densities and composite building units. [8]

Framework	Density / T 1000Å^{-3}	Composite building units
CHA	15·1	*d6r, cha-cage*
LTA	14·2	*d4r, sodalite-cage, lta-cage*
RHO	14·5	*d8r, lta-cage*
ABW	17·6	*abw-cage*
MOR	17·2	*mor-cage*

S8 Protonated MOR calculations

To discern whether our predictions can be extended to other ring systems, we examined the catalytically important MOR framework, which is made up from chains of linked five-membered rings forming alternating eight and twelve-ring channel systems in one direction. Unlike the frameworks discussed above in which all the T-sites are crystallographically indistinct, the MOR framework contains four distinct T-sites. For this reason, we only investigated the protonated form of the zeolite with two aluminiums at the nearest neighbour (NL) and next nearest neighbour positions (L) for each of the four T-sites, and in doing so we surveyed more than 800 different configurations of 2 Al per unit cell. Each configuration was optimized as 1:1:2 supercells using DFT (CP2K) at the PBE level of theory. The DFT data confirmed that even for this five-membered ring system, non-Löwensteinian aluminium ordering is the most thermodynamically stable framework configuration, where $\Delta E(NL_{global\ minimum} - L_{global\ minimum}) = 16{\cdot}1$ kJmol^{-1} per U.C., this energy difference is consistent with the trends in thermodynamic preference for NL structures and density observed for the other zeolites included in this work. The NL H-MOR global minimum structure is shown below.

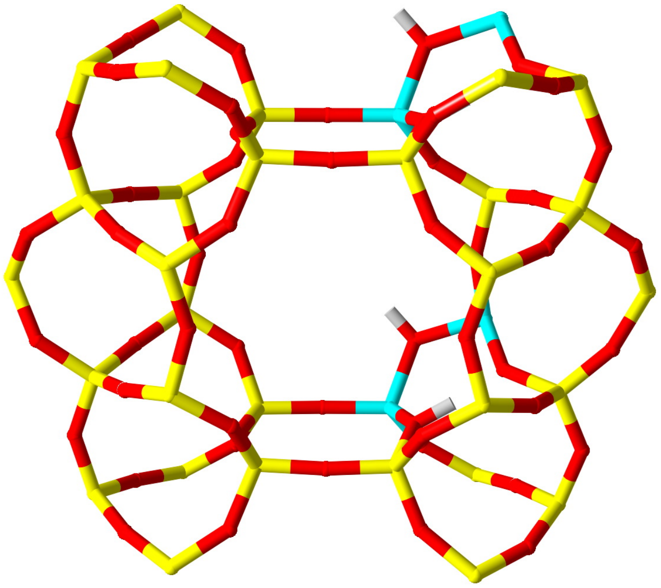

S9 Non-Löwensteinian global minimum structures according to DFT for RHO, LTA, ABW-type frameworks in their protonated forms with 2 Al per unit cell.
A) LTA B) RHO C) ABW

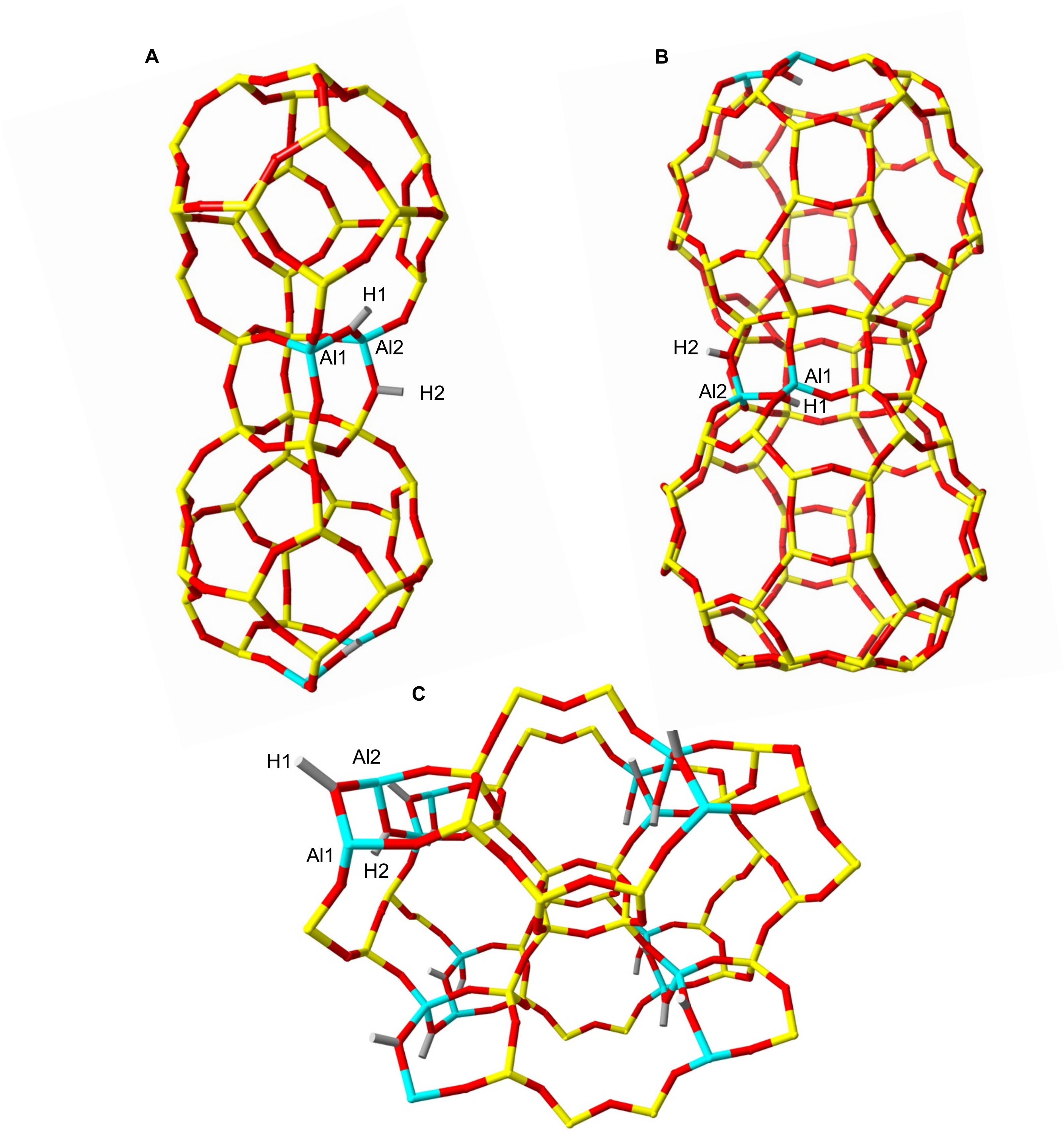

S10 SS MAS ^{29}Si NMR Data

Solid-state NMR calculations were performed for the L and NL 2 Al per unit cell global minimum structures using CASTEP (version 8.0)[5] with the CP2K optimized geometry because of the high computational cost of geometry optimisations of large systems associated with planewave basis sets. Our tests on a similar porous system, the UiO-66 metal-organic framework, showed that the difference in calculated NMR chemical shifts due to minor geometry differences between different DFT functionals and computational codes can indeed be safely neglected. All CASTEP calculations were performed using the PBE functional, on-the-fly pseudopotentials and planewave basis sets with a cutoff of 60 Ry, and a Monkhorst-Pack *k*-points grids of (3x3x3) were used to sample the Brillouin zone. The ^{29}Si chemical shifts, referenced to tetramethylsilane (TMS), are shown below.

The anticipated chemical shifts for different silica environments are as follows:[9]

4Si (0Al)	3Si (1Al)	2Si (2Al)	1Si (3Al)	0Si (4Al)
-100 to -115 ppm	-96 to -107 ppm	-91 to -100 ppm	-85 to -95 ppm	-80 to -91 ppm

Increasing the amount of aluminium bonded to the silica tetrahedra significantly decreases the negativity of the chemical shift (shifted downfield). This phenomenon is observed in our DFT predictions. The average chemical shift of 4Si in both frameworks is approximately -114 ppm, this increases to a maximum shift of -104.9 ppm for SiO_4 bonded to a single aluminium in the NL structure. Our predictions show that this method could not be used to characterize -Al-O-Al- at such a high Si/Al ratio. Firstly, the chemical shifts for all silica environments are far too similar, also any peaks that could be considered 'characteristic' of a nearby –Al-O(H)-Al- would be lost in background noise in the NMR spectrum.

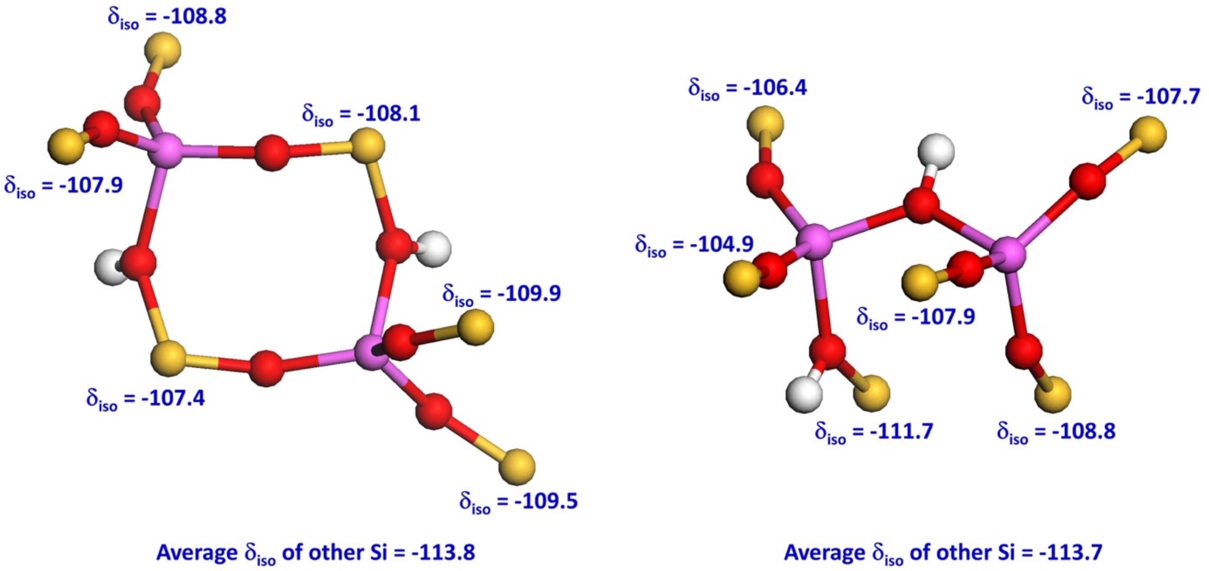

(referenced to TMS: 328.5039 ppm)

S11 Vibrational frequencies

Using CP2K, we predicted the vibrational frequencies of the L and NL 2 Al / unit cell H-SSZ-13 global minimum structures. The vibrational frequencies are shown below, the stretches indicative of a free hydroxyl bonded at –Si-O-Al- and -Al-O-Al- (3500 - 3700 cm^{-1}) are highlighted in grey. These stretches overlap with other broad stretches and would not be uniquely discernible.

Vibrational Frequencies / cm^{-1}

NL H-SSZ-13				L H-SSZ-13			
41.086	280.913	459.911	775.480	34.079	276.316	451.709	773.324
60.700	284.215	464.448	776.906	55.845	279.429	453.624	774.907
62.841	285.040	466.106	780.052	58.215	281.211	457.809	778.264
66.204	286.070	467.836	780.401	67.772	282.933	462.642	779.523
71.213	288.480	470.124	794.045	69.986	285.094	464.906	793.011
79.036	291.250	470.484	796.538	73.962	286.222	467.940	794.640
83.716	293.592	472.992	800.852	77.216	288.777	469.025	798.655
91.913	295.027	476.505	974.877	79.369	289.208	470.884	993.773
93.805	296.247	477.041	977.147	84.305	290.988	473.611	1002.309
97.372	297.325	479.247	998.464	91.581	293.718	476.569	1003.629
101.567	298.764	480.501	1001.794	95.905	296.944	478.007	1008.455
103.036	301.419	480.755	1005.069	99.897	297.771	480.059	1009.491
109.587	302.791	482.842	1008.935	104.715	299.632	481.547	1012.134
112.457	303.172	484.444	1013.073	107.956	302.274	483.072	1014.181
115.894	305.490	487.453	1015.120	109.689	303.286	486.479	1015.945
122.126	308.469	490.901	1017.229	114.726	306.703	487.946	1017.846
123.307	310.304	491.421	1019.178	117.631	306.916	490.320	1018.454
127.511	314.905	493.762	1020.337	120.582	309.430	493.185	1019.376
134.077	317.646	495.863	1021.235	128.715	310.100	497.700	1022.931
134.407	319.222	499.681	1022.248	131.484	314.976	501.279	1024.867
137.322	320.221	506.260	1024.148	134.693	316.073	505.999	1027.613
142.557	324.034	509.776	1024.340	137.829	318.730	507.753	1028.312
145.870	326.486	511.175	1027.749	138.905	319.934	509.569	1029.497
147.035	330.468	515.541	1029.806	142.454	326.546	511.889	1031.092
147.733	331.858	536.236	1030.197	144.373	328.034	528.920	1032.354
151.114	333.907	544.018	1031.939	144.928	329.369	531.849	1033.877
152.361	337.729	551.341	1032.664	150.270	331.673	546.898	1034.714
156.749	338.623	563.546	1035.630	154.458	332.887	553.237	1036.601
158.904	340.575	568.779	1036.356	156.840	334.536	568.751	1039.284
160.488	341.975	583.620	1037.189	158.086	337.647	586.200	1039.398
161.486	342.371	591.713	1038.148	161.620	338.406	588.652	1040.573
165.783	344.140	593.259	1038.653	164.384	339.932	592.987	1041.621
166.708	347.491	596.488	1042.923	166.364	342.812	594.907	1044.496
170.959	350.620	598.476	1043.823	168.419	347.192	598.416	1045.304
172.948	352.842	601.131	1044.487	169.194	347.824	598.575	1047.497
174.223	354.989	601.522	1047.496	173.004	350.606	600.278	1047.930
177.640	358.478	608.647	1049.074	176.228	352.831	601.513	1051.638
178.923	360.613	612.718	1050.657	176.860	353.919	607.803	1053.262
182.899	361.603	614.399	1052.260	179.789	361.705	608.873	1054.064
183.027	363.761	616.432	1055.648	184.165	363.032	617.486	1055.753
184.783	367.420	620.433	1059.292	185.135	364.830	619.851	1056.985
185.955	371.459	624.156	1066.223	185.732	367.279	620.922	1073.847
188.620	373.127	627.769	1073.359	187.387	368.186	623.672	1082.761
190.671	374.184	637.823	1083.783	188.126	371.972	642.725	1087.536
192.691	376.490	652.838	1103.831	190.826	372.989	652.676	1099.736
193.738	377.708	666.989	1111.319	191.002	373.270	656.263	1110.110
194.571	379.022	677.882	1121.494	193.120	374.284	672.431	1116.757
195.994	380.609	680.600	1122.121	194.137	377.214	676.902	1117.905
196.682	382.328	685.354	1123.747	194.497	378.374	678.813	1121.236
198.895	383.619	686.535	1125.932	197.730	380.332	682.719	1126.825
200.963	385.723	693.316	1128.760	198.988	381.666	693.006	1128.819

202.850	386.332	697.145	1131.447	202.576	382.311	708.745	1129.863
203.654	387.263	713.096	1135.988	203.254	385.485	722.359	1134.511
204.135	388.917	730.237	1137.010	206.637	385.882	728.002	1135.050
207.644	391.012	734.399	1138.314	208.239	387.447	733.763	1140.596
209.803	391.441	738.851	1142.604	208.841	387.864	734.614	1142.541
213.123	394.794	742.477	1143.669	214.696	389.946	740.692	1146.420
216.380	395.470	746.615	1145.350	219.292	392.472	743.545	1147.640
221.024	397.282	748.056	1148.473	219.734	393.165	748.788	1148.864
224.110	399.088	750.353	1150.856	220.819	393.821	749.008	1152.122
226.453	401.381	751.393	1151.481	221.968	398.271	749.974	1152.560
228.693	402.226	752.215	1155.128	226.289	399.384	751.049	1156.436
230.187	403.995	752.989	1156.439	229.192	401.921	752.203	1158.169
232.893	405.128	754.186	1158.231	232.483	403.626	752.689	1159.746
235.102	407.200	754.979	1158.909	238.339	405.894	753.368	1163.000
245.470	413.514	756.073	1161.212	239.145	413.223	754.148	1164.140
247.639	413.935	756.288	1163.527	244.171	414.555	756.003	1165.520
250.655	416.097	757.296	1164.708	248.343	417.860	757.088	1165.981
252.351	418.391	759.423	1167.186	251.621	419.769	757.548	1167.974
258.163	421.317	760.947	1169.325	253.637	423.265	757.887	1170.740
261.616	422.868	763.969	1171.032	254.586	423.590	760.133	1172.795
263.343	425.553	765.144	1172.328	256.397	427.084	761.962	1174.619
264.785	428.809	766.028	1175.551	260.969	431.194	763.313	1176.196
266.483	432.927	766.922	1181.557	261.894	434.051	765.538	1177.260
268.482	436.148	767.268	1184.349	263.797	435.187	766.050	1179.158
270.691	437.957	767.892	1187.353	266.189	438.149	767.873	1181.042
272.142	438.603	769.125	1191.960	267.723	438.283	768.272	1185.312
273.041	438.751	769.933	1203.460	269.031	439.838	768.527	1190.400
275.187	442.348	770.510	1205.895	270.299	441.582	770.129	1201.193
276.693	445.236	772.746	3665.621	272.041	443.347	770.755	3696.139
276.938	448.423	773.513	3698.982	273.308	448.831	771.415	3699.446
277.645	454.582	774.920		275.201	449.963	772.230	

S12 Zero Point Energy Calculations and Thermodynamics Calculations

Using the IR data (S7) we calculated the zero point energies for the 2 Al/ unit cell (Si/Al = 17) H-SSZ-13 L and NL global minima structures, obtained from DFT (CP2K) at the PBE level of theory, with the TZV2P basis set. Zero point energy = ZPE.

ZPE for NL = 1123.3 kJ mol^{-1}
ZPE for L = 1119.9 kJ mol^{-1}
Δ ZPE (NL-L) = 3.43 kJ mol^{-1}
ΔE (NL-L) = -14.21 kJ mol^{-1}
ΔE (NL-L) + ZPE = -10.78 kJ mol^{-1}

We then compared these results with data obtained using CASTEP for the smaller 2 Al per unit cell 12 T-site rhombohedral unit cell, (Si/Al = 5).

ZPE for NL = 398.4 kJ mol^{-1}
ZPE for L = 398.20 kJ mol^{-1}
Δ ZPE (NL-L) = 0.20 kJ mol^{-1}
ΔE (NL-L) = -9.06 kJ mol^{-1}
ΔE (NL-L) + ZPE = -8.86 kJ mol^{-1}

The absolute zero point energy difference between the CASTEP data with a 10 T atom unit cell are slightly lower than that obtained using CP2K for the larger 36 T atom unit cell. This is due to a combination of the lower Si/Al ratio and differences in the pseudopotential and basis sets used. The key point is that the enthalpy difference for NL-L still favours NL by ~10 kJ /mol when corrections for the ZPE are considered.

CASTEP was used to carry out an assessment of the free difference, taking account of the vibrational entropy differences within the static, harmonic approximation with a cutoff of 800 eV. Phonon frequencies were sampled at 4 different k-points in the Brillouin zone.

According to CASTEP, the internal energy difference between the Si/Al = 5 global minimum configurations is ΔE(NL-L) = -9.06 kJ/mol. The Helmholtz free-energy difference between configurations as a function of temperature is reported below. The NL configuration is marginally stabilised with respect to L with increasing temperature.

TEMPERATURE / K	HELMHOLTZ FREE ENERGY DIFFERENCE (NL-L) INCLUDING ZERO POINT ENERGY /$kJmol^{-1}$
0	-8.86
37	-8.89
73	-8.99
110	-9.10
147	-9.21
183	-9.32
220	-9.43
257	-9.53
293	-9.63
330	-9.73
367	-9.83
403	-9.93
440	-10.03
476	-10.12
513	-10.22
550	-10.32
586	-10.42
623	-10.51
660	-10.61
696	-10.71

S13 CP2K example input file

A typical CP2K input file is given below. All geometries and CP2K inputs available on request.

```
&GLOBAL
   PRINT_LEVEL  MEDIUM
   PROJECT_NAME EXAMPLE
   RUN_TYPE  CELL_OPT
   FLUSH_SHOULD_FLUSH  T
 &END GLOBAL
 &MOTION
   &GEO_OPT
     TYPE  MINIMIZATION
     OPTIMIZER  LBFGS
     MAX_ITER  3000
     MAX_DR     2.9999999999999997E-04
     MAX_FORCE     4.5000000000000003E-05
     RMS_DR     1.4999999999999999E-04
     RMS_FORCE     3.0000000000000001E-05
     &CG
       MAX_STEEP_STEPS  0
       &LINE_SEARCH
         TYPE  2PNT
       &END LINE_SEARCH
     &END CG
   &END GEO_OPT
   &CELL_OPT
     OPTIMIZER  CG
     MAX_ITER  1000
     MAX_DR     3.0000000000000001E-03
     MAX_FORCE     4.4999999999999999E-04
     RMS_DR     1.5000000000000000E-03
     RMS_FORCE     2.9999999999999997E-04
     STEP_START_VAL  0
     TYPE  DIRECT_CELL_OPT
     EXTERNAL_PRESSURE     1.0000000000000000E+00
     KEEP_ANGLES  T
     PRESSURE_TOLERANCE     1.0000000000000000E+01
     &CG
       MAX_STEEP_STEPS  0
       RESTART_LIMIT     9.4999999999999996E-01
       &LINE_SEARCH
         TYPE  2PNT
       &END LINE_SEARCH
     &END CG
     &PRINT
       &PROGRAM_RUN_INFO  MEDIUM
       &END PROGRAM_RUN_INFO
       &CELL  MEDIUM
       &END CELL
     &END PRINT
   &END CELL_OPT
 &END MOTION
 &FORCE_EVAL
   METHOD  QS
   STRESS_TENSOR  ANALYTICAL
   &DFT
  BASIS_SET_FILE_NAME ./GTH_BASIS_SETS
  POTENTIAL_FILE_NAME ./POTENTIAL
     CHARGE  0
     &SCF
       MAX_SCF  20
       EPS_SCF     9.9999999999999995E-08
       SCF_GUESS  ATOMIC
       &OT  T
         MINIMIZER  DIIS
         PRECONDITIONER  FULL_ALL
         ENERGY_GAP     1.0000000000000000E-03
       &END OT
       &OUTER_SCF  T
         EPS_SCF     9.9999999999999995E-08
       &END OUTER_SCF
     &END SCF
     &QS
       EPS_DEFAULT     9.9999999999999998E-13
     &END QS
     &MGRID
       CUTOFF     6.5000000000000000E+02
     &END MGRID
     &XC
```

```
      DENSITY_CUTOFF     1.0000000000000000E-10
      GRADIENT_CUTOFF     1.0000000000000000E-10
      TAU_CUTOFF     1.0000000000000000E-10
      &XC_FUNCTIONAL  NO_SHORTCUT
        &PBE  T
          PARAMETRIZATION  ORIG
        &END PBE
      &END XC_FUNCTIONAL
    &END XC
    &POISSON
      POISSON_SOLVER  PERIODIC
      PERIODIC  XYZ
    &END POISSON
  &END DFT
  &SUBSYS
    &CELL
### ABC according to zeolite framework structure ###
      &CELL_REF
### ABCref 5% > ABC ###
      &END CELL_REF
    &END CELL
    &COORD
### zeolite crystal structure ###
    &END COORD
    &KIND Si
      BASIS_SET TZV2P-GTH
      POTENTIAL GTH-PBE-q4
    &END KIND
    &KIND O
      BASIS_SET TZV2P-GTH
      POTENTIAL GTH-PBE-q6
    &END KIND
    &KIND Al
      BASIS_SET TZV2P-GTH
      POTENTIAL GTH-PBE-q3
    &END KIND
    &KIND H
      BASIS_SET TZV2P-GTH
      POTENTIAL GTH-PBE-q1
    &END KIND
    &KIND Na
      BASIS_SET TZV2P-GTH
      POTENTIAL GTH-PBE-q9
    &END KIND
    &TOPOLOGY
      NUMBER_OF_ATOMS  ### according to zeolite framework structure
      MULTIPLE_UNIT_CELL  1 1 1
    &END TOPOLOGY
  &END SUBSYS
 &END FORCE_EVAL
```

S14 Coarse-grain sifting for potential low-energy structures using GULP

Investigating high-silica SSZ-13, 2 Al / unit cell, involved substituting a single uninodal Si T-site with Al and then sequentially substituting the remaining Si T-sites with a second Al, as described in the main body of the text. However, for lower silica frameworks, tending to a Si/Al of unity becomes increasingly more complex with each additional Al introduced to the framework. Introducing a third Al into the H-SSZ-13 framework results in 487, 344 possible combinations of 3 Al/ unit cell across the 36 T-sites, each with an associated proton at a neighbouring oxygen site. Manually constructing each of these models is rather time consuming, and fully optimising each model at the DFT level of theory is very compute intensive.

We attempted to reduce computational expense by utilising force field methods to determine potential low-energy structures, before fully optimising the most stable structures quantum mechanically using CP2K. Our method involved substituting the smaller 12 T-site, rhombohedral CHA unit-cell with a single aluminium, screening with GULP[10,11], and subsequently substituting the 50 lowest energy structures with a second aluminium. This methodology was repeated and continued until six of the 12 T-sites were occupied with Al (Si/Al = 1). We used both the Catlow potential and related modifications[12] – widely used in zeolite science, and the Clay Force Field (clayFF)[13], with a range of framework constraints, including constant pressure and constant volume calculations. However, disappointingly and despite extenisve re-fitting, we found only weak correlation (a coefficient of ~0.1) between the force field models and the DFT (PBE) data previously obtained for the high silica SSZ-13 case. Critically, the global minima differed and energy differences between configurations were in large absolute and relative error with respect to the DFT data. Despite extensive attempted optimisations of both forcefields, we were unable to obtain sufficient correlation between the force field and DFT data to allow us to use the forcefields as a pre-filter before DFT. In the light of this, we adopted the purely QM based method of stepwise substitution in the global minima structure described in the main body of the text.

S15 References

1. J. P. Perdew, K. Burke, M. Ernzerhof, *Phys. Rev. Lett.* **78**, 1396–1396 (1997).
2. Y. Zhang, W. Yang, *Phys. Rev. Lett.* **80**, 890–890 (1998).
3. A. D. Becke, *Phys. Rev. A*. **38**, 3098–3100 (1988).
4. C. Lee, W. Yang, R. G. Parr, *Phys. Rev. B*. **37**, 785–789 (1988).
5. S. J. Clark *et al.*, *Zeitschrift fuer Krist.* **220**, 567–570 (2005).
6. M. Guidon, J. Hutter, J. VandeVondele, *J. Chem. Theory Comput.* **6**, 2348–2364 (2010).
7. C. Adamo, V. Barone, *J. Chem. Phys.* **110**, 6158 (1999).
8. C. Baerlocher, L. . McCusker, D. H. Olson, *Atlas of Zeolite Framework Types* (sixth edit., 2007).
9. J. Klinowski, *Prog. Nucl. Magn. Reson. Spectrosc.* **16**, 237–309 (1984).
10. J. D. Gale, *J. Chem. Soc. Faraday Trans.* **93**, 629–637 (1997).
11. J. D. Gale, A. L. Rohl, *Mol. Simul.* **29**, 291–341 (2003).
12. M. J. Sanders, M. Leslie, C. R. A. Catlow, *J. Chem. Soc. Chem. Commun.* **19**, 1271 (1984).
13. R. T. Cygan, J.-J. Liang, A. G. Kalinichev, *J. Phys. Chem. B*. **108**, 1255–1266 (2004).
